# The transporter and permeability interactions of asymmetric dimethylarginine (ADMA) and L-arginine with the human blood–brain barrier *in vitro*

**DOI:** 10.1016/j.brainres.2016.07.026

**Published:** 2016-10-01

**Authors:** Christopher P. Watson, Evangelos Pazarentzos, Mehmet Fidanboylu, Beatriz Padilla, Rachel Brown, Sarah A. Thomas

**Affiliations:** a King's College London, Institute of Pharmaceutical Science, Waterloo, London, UK; b Imperial College London, Experimental Medicine and Toxicology Section, Division of Experimental Medicine, London, UK

**Keywords:** ADMA, asymmetric dimethylarginine, BBB, blood-brain barrier, CAA, cationic amino acids, CAT-1, cationic amino acid transporter 1, DDAH, N^G^, N^G^-dimethylarginine dimethylaminohydrolase, GTPases, guanosine triphosphatases, IFN-γ, interferon gamma, LAT, large neutral amino acid transporters, L-NIO, N-iminoethyl-L-ornithine, MATE1, multidrug and toxin exclusion protein 1, NO, nitric oxide, NOS, nitric oxide synthase, OCT, organic cation transporter, OSPC, octanol-saline partition coefficient, PRMTs, protein-arginine methyltransferases, ROS, reactive oxygen species, TNF-α, tumour necrosis factor alpha, Cationic amino acid transporter 1, Nitric oxide, Guanosine triphosphatases (GTPases), Cerebral microvasculature

## Abstract

The blood–brain barrier (BBB) is a biological firewall that carefully regulates the cerebral microenvironment by acting as a physical, metabolic and transport barrier. This selectively permeable interface was modelled using the immortalised human cerebral microvascular endothelial cell line (hCMEC/D3) to investigate interactions with the cationic amino acid (CAA) L-arginine, the precursor for nitric oxide (NO), and with asymmetric dimethylarginine (ADMA), an endogenously derived analogue of L-arginine that potently inhibits NO production. The transport mechanisms utilised by L-arginine are known but they are not fully understood for ADMA, particularly at the BBB. This is of clinical significance giving the emerging role of ADMA in many brain and cerebrovascular diseases and its potential as a therapeutic target. We discovered that high concentrations of ADMA could induce endothelial dysfunction in the hCMEC/D3s BBB permeability model, leading to an increase in paracellular permeability to the paracellular marker FITC-dextran (40 kDa). We also investigated interactions of ADMA with a variety of transport mechanisms, comparing the data with L-arginine interactions. Both molecules are able to utilise the CAA transport system y^+^. Furthermore, the expression of CAT-1, the best known protein from this group, was confirmed in the hCMEC/D3s. It is likely that influx systems, such as y^+^L and b^0,+^, have an important physiological role in ADMA transport at the BBB. These data are not only important with regards to the brain, but apply to other microvascular endothelia where ADMA is a major area of investigation.

## Highlights

• ADMA interacts with a variety of transporters at the blood-brain barrier.
• These included cationic amino acid transporters, including CAT-1.
• Human blood-brain barrier endothelial cells express CAT-1.
• ADMA at high concentrations can disrupt the blood-brain barrier.
• This disruption is not linked to increased ROS at the blood-brain barrier.

## 1 Introduction

ADMA is an endogenously derived analogue of the CAA, L-arginine, and is produced during routine proteolysis of methylated arginine residues on proteins by a group of enzymes called protein-arginine methyltransferases (PRMTs) (Palm et al., 2007). Whereas L-arginine is a substrate for nitric oxide synthases (NOS) which catalyse the oxidation of L-arginine to L-citrulline and nitric oxide (NO) (using NADPH and O_2_ as co-substrates), ADMA acts as a potent inhibitor of NO production and inhibits all NOS isoforms by competing with L-arginine for the NOS binding site (Vallance et al., 1992, Leiper and Vallance, 1999, Tran et al., 2003, Siroen et al., 2006, Kittel and Maas, 2014).

Because of its potency as an inhibitor of NO, ADMA has been implicated in a wide variety of pathophysiological conditions throughout the body including hypercholesterolemia, hyperhomocysteinemia and hypertriglyceridemia (Boger et al., 1998, Lundman et al., 2001, Sydow et al., 2003); a host of cardiovascular conditions (Boger, 2003, Boger, 2004, Perticone et al., 2005); neuroinflammatory and neurodegenerative diseases including Alzheimer's disease (Selley, 2003, Arlt et al., 2008); cerebrovascular diseases such as stroke (Yoo and Lee, 2001, Brouns et al., 2009); microangiopathy-related cerebral damage (Notsu et al., 2009), and recently, intrauterine foetal growth restriction (Laskowska et al., 2014).

Given these disease implications, it is somewhat surprising that the basic cellular transport mechanisms of ADMA remain understudied. The main reason is the structural similarity of ADMA to L-arginine, suggesting that both molecules share transport mechanisms, although data *directly* looking at ADMA membrane transport in vascular endothelial cells is lacking. Recent work by Strobel et al. showed that ADMA is able to utilise CAT2 (2A and 2B), organic cation transporter 2 (OCT2) and multidrug and toxin exclusion protein 1 (MATE1), albeit in a HEK239 cell model overexpressing these transporters and not vascular endothelial cells. They showed that the transport kinetics of CAT2A, CAT2B, and OCT2 indicate a low affinity, high capacity transport for ADMA (Strobel et al., 2013). Interestingly, a study by Ohtsuki et al. (2013) could not detect the expression of MATE1 and OCT2 membrane proteins in hCMEC/D3 cells. In addition, the transport of ADMA at the blood-brain barrier (BBB) has yet to be investigated despite the emerging role of the molecule in brain and cerebrovascular pathologies and the importance of the BBB as a dynamic interface between the brain and blood (Abbott et al., 2006). The transport mechanisms for L-arginine have been described at the bovine BBB: the principle L-arginine transporters here are CAT1, CAT2B and CAT3, which are all members of system y^+^ (O'kane et al., 2006). Interestingly, other data have implicated the system y^+^ large neutral amino acid transporters (y^+^LAT) y^+^LAT1 and y^+^LAT2 in human *in vitro* BBB models (Carl et al., 2010).

One of the aims of this study was to test the hypothesis that ADMA has a similar transport profile to L-arginine in human brain endothelial cells. A major issue with BBB research (as with most other fields) is that the wide variety of models in use can make it difficult to relate and compare data, particularly between species. For example, much of the CAA transport work has utilised single cloned transporters expressed in *Xenopus laevis* oocytes and this is unlikely to give an accurate indication of the situation in mammalian systems where multiple transporters are present.

The hCMEC/D3 cell line is a commercially available, easily grown and transferable population of human microvascular CEC that stably maintains a normal BBB phenotype. These include expression of tight junction proteins, polarized expression of multiple ABC/SLC transporters and restrictive permeability. It has been used in over 100 different published studies of the BBB (Weksler et al., 2013).

We thus compared the transport of ADMA and L-arginine in an accumulation model format using this well-established hCMEC/D3 human BBB model (Watson et al., 2012).

We also investigated the association between ADMA concentration and endothelial dysfunction. Such associations have been made in a variety of pathological conditions including chronic heart failure, renal failure, and a variety of diabetic complications such as renin-angiotensin system mediated diabetic retinopathy (Boger et al., 1998, Leiper et al., 2007, Chen et al., 2009, Chen et al., 2011). ADMA and its metabolising enzyme, N^G^, N^G^-dimethylarginine dimethylaminohydrolase (DDAH), have also recently been implicated as regulators of pulmonary endothelial barrier function through the modulation of small guanosine triphosphatases (GTPases). This has been demonstrated in both *in vitro* and *in vivo* with mouse models (Wojciak-Stothard et al., 2007, Wojciak-Stothard et al., 2009). ADMA has also been heavily implicated in the production of reactive oxygen species (ROS) *via* uncoupling of endothelial NOS, causing the enzyme to produce superoxides and not NO (Sydow and Munzel, 2003, Chen et al., 2011).

One of the hallmarks of endothelial dysfunction is an increase in leakiness *via* the paracellular route. Whereas the abovementioned studies investigated the effects of ADMA on non-cerebral vasculature, no group has yet studied ADMA and BBB dysfunction. This is of particular importance because not only is BBB integrity paramount for maintaining homeostasis within the cerebral microenvironment, but ADMA has also been implicated in endothelial dysfunction in brain and cerebrovascular disorders such as ischemic stroke (Scherbakov et al., 2012).

Therefore, we investigated the effects of ADMA on the integrity of the human BBB *in vitro* using the hCMEC/D3 cell line in a paracellular permeability model format. Other *in vitro* studies have only investigated ADMA in cerebral endothelial dysfunction at high concentrations, so we decided to look at a range covering the physiological and pathophysiological concentrations of ADMA. We hypothesised that increased concentrations of ADMA would lead to increased BBB permeability, especially at concentrations in line with previous non-BBB studies, but did not rule out the possibility that lower concentrations may also induce permeability changes.

## 2 Results

### 2.1 Octanol-saline partition coefficient (OSPC) of [^3^H]l-arginine and [^3^H]ADMA

An OSPC was performed on all radiolabelled compounds to assess their relative lipophilicity which would give an indication of their potential to cross cell membranes by passive diffusion and accumulate in the cells. The mean OSPC values for [^3^H]L-arginine (MW 174.2 g/mol) and [^3^H]ADMA (MW 202.25 g/mol) were 0.0015±0.0002 and 0.0023±0.0001 respectively (n=3). These values are statistically significantly different from each other (*p*<0.01). Our group previously performed OSPC analysis on [^14^C]sucrose (MW 342.3 g/mol; n=3) obtaining a mean value of 0.0004±0.00001, which was significantly lower when compared to [^3^H]L-arginine and [^3^H]ADMA (both *p*<0.001).

### 2.2 [^3^H]L-arginine and [^3^H]ADMA accumulation comparison

**Fig. 1:**
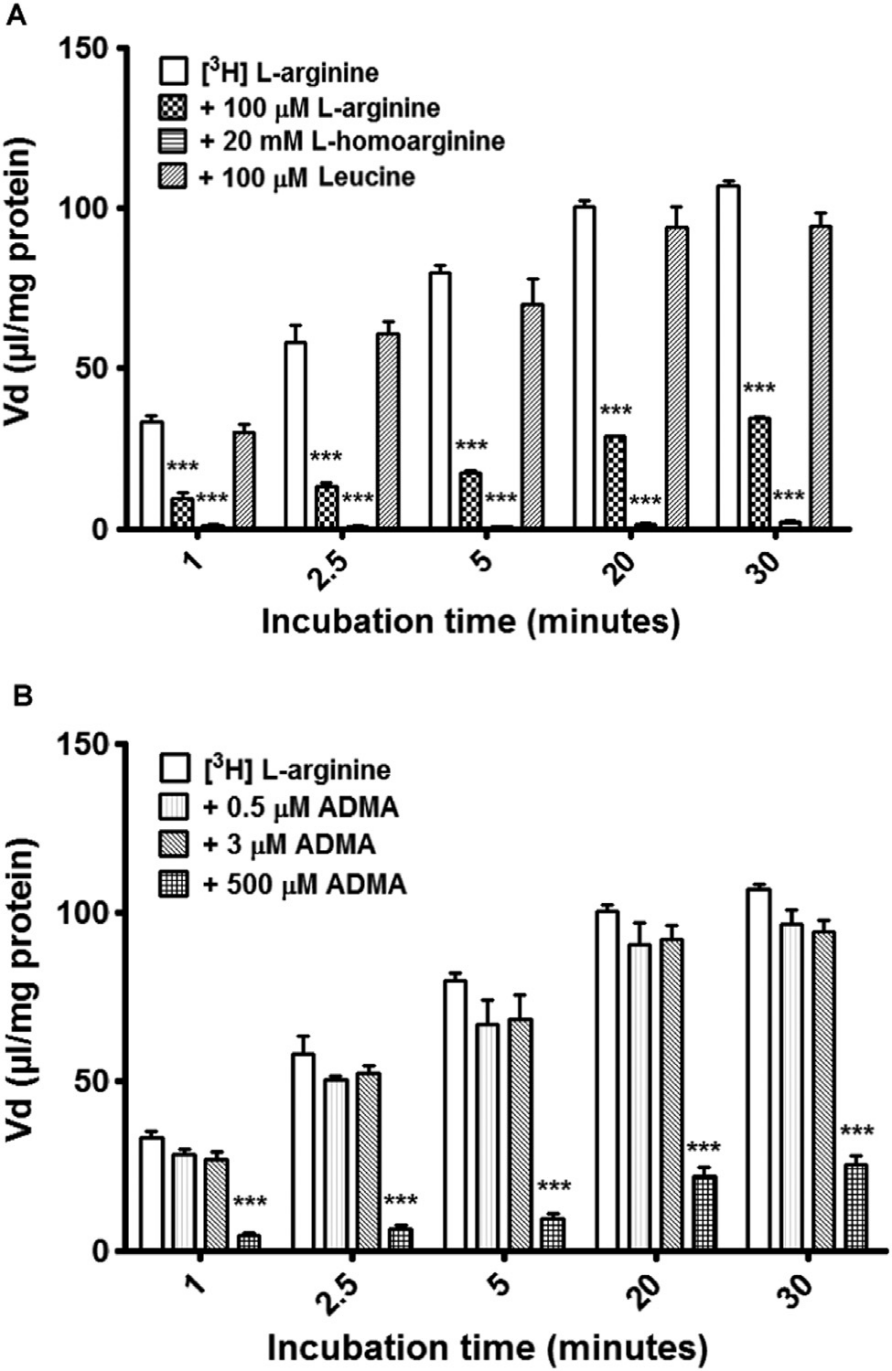
Accumulation of [^3^H]L-arginine in hCMEC/D3 cells. (A) To understand the roles CAA transporters played in the transport and subsequent accumulation of [^3^H]L-arginine, self-inhibition and the addition of known AA transporter interacting molecules was performed in an accumulation model using confluent hCMEC/D3s. Control L-arginine values were compared to treatments. ***p<0.001. (B) To investigate the competitive effects of unlabelled ADMA on [^3^H]L-arginine accumulation, 0.5 µM, 3 µM and 500 µM ADMA were added individually to accumulation buffer. ***p<0.001. All [^3^H]L-arginine data are corrected for [^14^C]sucrose and protein content (with [^14^C]sucrose corrected for protein only) and expressed as means±SEM, n=6 plates with 6 replicates per plate.

**Fig. 2:**
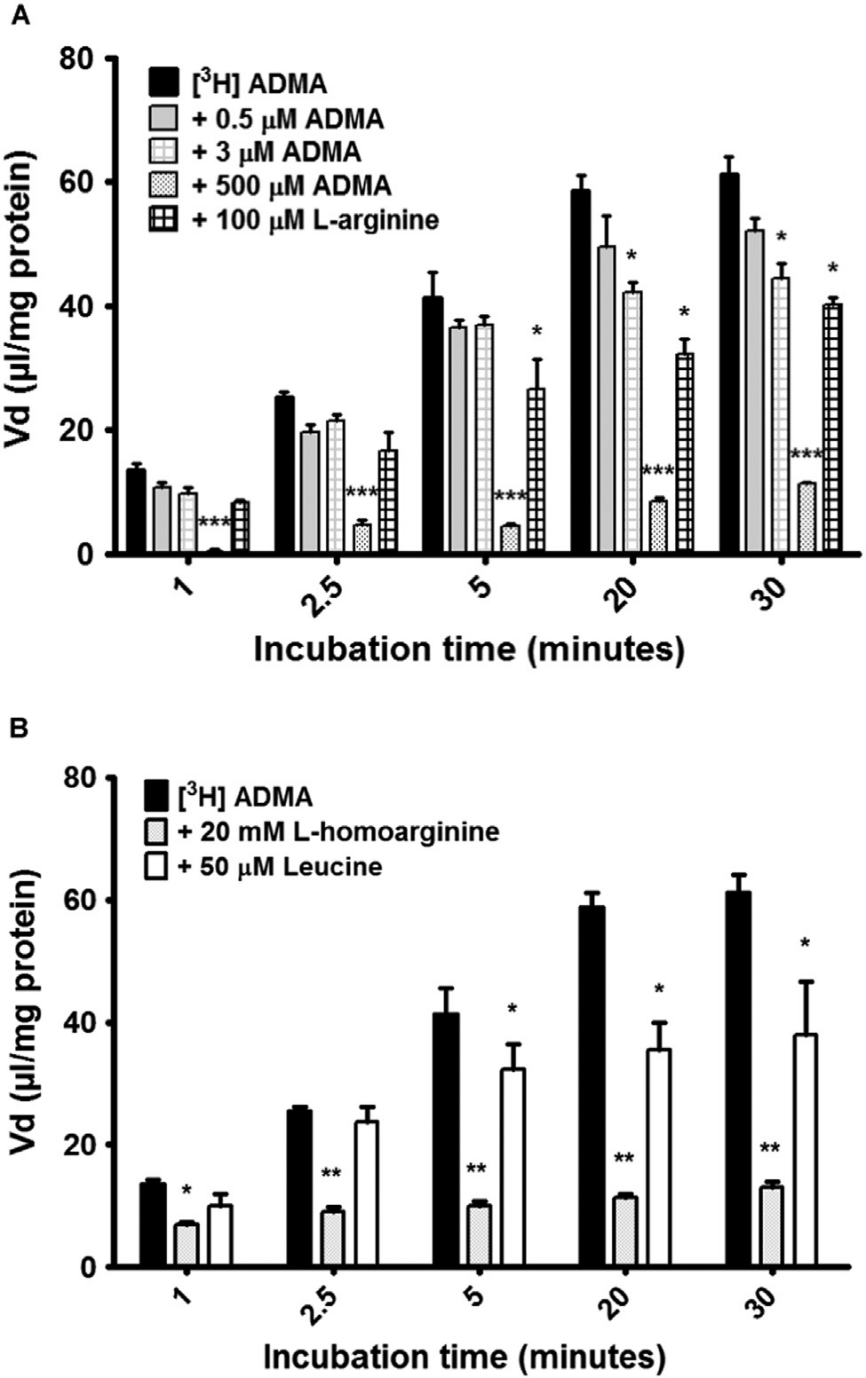
Accumulation of [^3^H]ADMA in hCMEC/D3 cells. (A) To investigate self and competitive inhibition on [^3^H]ADMA transport and accumulation, 0.5 µM, 3 µM and 500 µM unlabelled ADMA and 100 µM L-arginine were added individually to the accumulation buffer. *p<0.05,***p<0.001. (B) To understand the roles CAA transporters played in the transport and subsequent accumulation of [^3^H]ADMA, the addition of known AA transporter interacting molecules was performed in an accumulation model using confluent hCMEC/D3s. *p<0.05,**p<0.01. All [^3^H]ADMA data are corrected for protein content and [^14^C]sucrose (with [^14^C]sucrose corrected for protein only) and expressed as means±SEM, n=5 (A) or 4–6 (B) plates with 6 replicates per plate.

The values for [^14^C]sucrose and protein corrected [^3^H]L-arginine and [^3^H]ADMA accumulation in the hCMEC/D3 cells are shown in Fig. 1, Fig. 2, respectively. The accumulation of [^3^H]ADMA (38 nM) in the hCMEC/D3 cells increased in a time-dependent manner from a Vd of 13.58±0.66 μl/mg protein at 1 min, to a Vd of 61.24±2.92 μl/mg protein at 30 min. In a similar manner, the accumulation of [^3^H]L-arginine (7 nM) increased in a time-dependent manner from a Vd of 33.75±1.70 μl/mg protein at 1 min, to a Vd of 106.79±1.73 μl/mg protein at 30 min. At all time points, [^3^H]L-arginine Vd values were significantly higher than those for [^3^H]ADMA (*p*<0.001 at all time points).

### 2.3 Accumulation of [^3^H]L-arginine – role of CAA transporters

To assess the roles of CAA transporters on the transport and accumulation of [^3^H]L-arginine in the hCMEC/D3s, 100 µM unlabelled L-arginine, 20 mM L-homoarginine and 100 µM L-leucine were added individually to the accumulation buffer alongside [^3^H]L-arginine and [^14^C]sucrose, and compared to appropriate controls.

The control Vd values (μl/mg of protein) for [^3^H]L-arginine were 33.75 at 1 min, 57.99 at 2.5 min, 79.84 at 5 min, 100.30 at 20 min and 106.79 at 30 min. The addition of 100 µM unlabelled L-arginine caused a marked decrease in the accumulation of [^3^H]L-arginine (*p*<0.001), with an average percentage decrease across the time points of 73% (Fig. 1A). Accumulation was almost completely inhibited (with an approximate decrease of 98% observed across all time points) with the addition of 20 mM L-homoarginine (*p*<0.001). The addition of 100 µM L-leucine to the accumulation buffer caused no significant changes in [^3^H]L-arginine accumulation.

To investigate the possible competitive effect of ADMA on the influx and accumulation of [^3^H]L-arginine, 0.5 µM, 3 µM, and 500 µM unlabelled ADMA were added individually to the accumulation buffer. When 0.5 µM and 3 µM ADMA was added, there were no significant differences in accumulation when compared to [^3^H]L-arginine controls. However, an average decrease in [^3^H]L-arginine accumulation of 83% across the time points was seen with the addition of 500 µM ADMA (*p*<0.001) when compared to controls (Fig. 1B).

### 2.4 Accumulation of [^3^H]ADMA – role of CAA transporters

To elucidate the transport mechanisms utilised by ADMA in the hCMEC/D3s, a series of accumulation experiments similar to those used for [^3^H]L-arginine were designed to assess the effects of self-inhibition, competitive inhibition and CAA transporters on the transport and resulting accumulation of [^3^H]ADMA and [^14^C]sucrose.

The control Vd values (μl/mg of protein) for [^3^H]ADMA were 13.58 at 1 min, 25.36 at 2.5 min, 41.32 at 5 min, 58.76 at 20 min and 61.24 at 30 min. To investigate self- and cross-inhibition of [^3^H]ADMA transport and accumulation, 0.5 µM, 3 µM and 500 µM unlabelled ADMA as well as 100 µM L-arginine were added individually to the accumulation buffer which also contained [^3^H]ADMA and [^14^C]sucrose. 0.5 µM ADMA caused a decrease in the accumulation of [^3^H]ADMA, but this failed to attain statistical significance. In contrast, 3 µM caused significant decreases in [^3^H]ADMA accumulation at 20 min and 30 min (*p*<0.05*,* Fig. 2A). A decrease in [^3^H]ADMA accumulation of 87% was observed across all time points on the addition of 500 µM ADMA (*p*<0.001). 100 µM L-arginine also caused an average decrease of 36% across time points, but statistical significance was only apparent after 5 min, 20 min and 30 min (*p*<0.05 for each comparison).

The influence of CAA transporters on [^3^H]ADMA and [^14^C]sucrose was initially determined using 20 mM L-homoarginine. The addition of 20 mM L-homoarginine caused a large decrease in accumulation of [^3^H]ADMA at all time points, which gained higher significance as the incubation time increased (Fig. 2B). The percentage decreases in Vd induced by L-homoarginine were 47% for 1 min, 64% for 2.5 min, 75% for 5 min, 8%1 for 20 min and 76% for 30 min. The addition of 100 µM L-leucine saw significant decreases in [^3^H]ADMA accumulation from 5 min (21% decrease), to 20 min (40% decrease) and 30 min (38% decrease, all *p*<0.05).

### 2.5 Accumulation of [^14^C]sucrose

Sucrose was used throughout the transport assays as a marker for unspecific binding and extracellular space. There were no significant changes in the accumulation of [^14^C]sucrose from baseline values in any transport studies in this investigation (data not shown).

### 2.6 Expression of CAT-1 in hCMEC/D3 cells

**Fig. 3:**
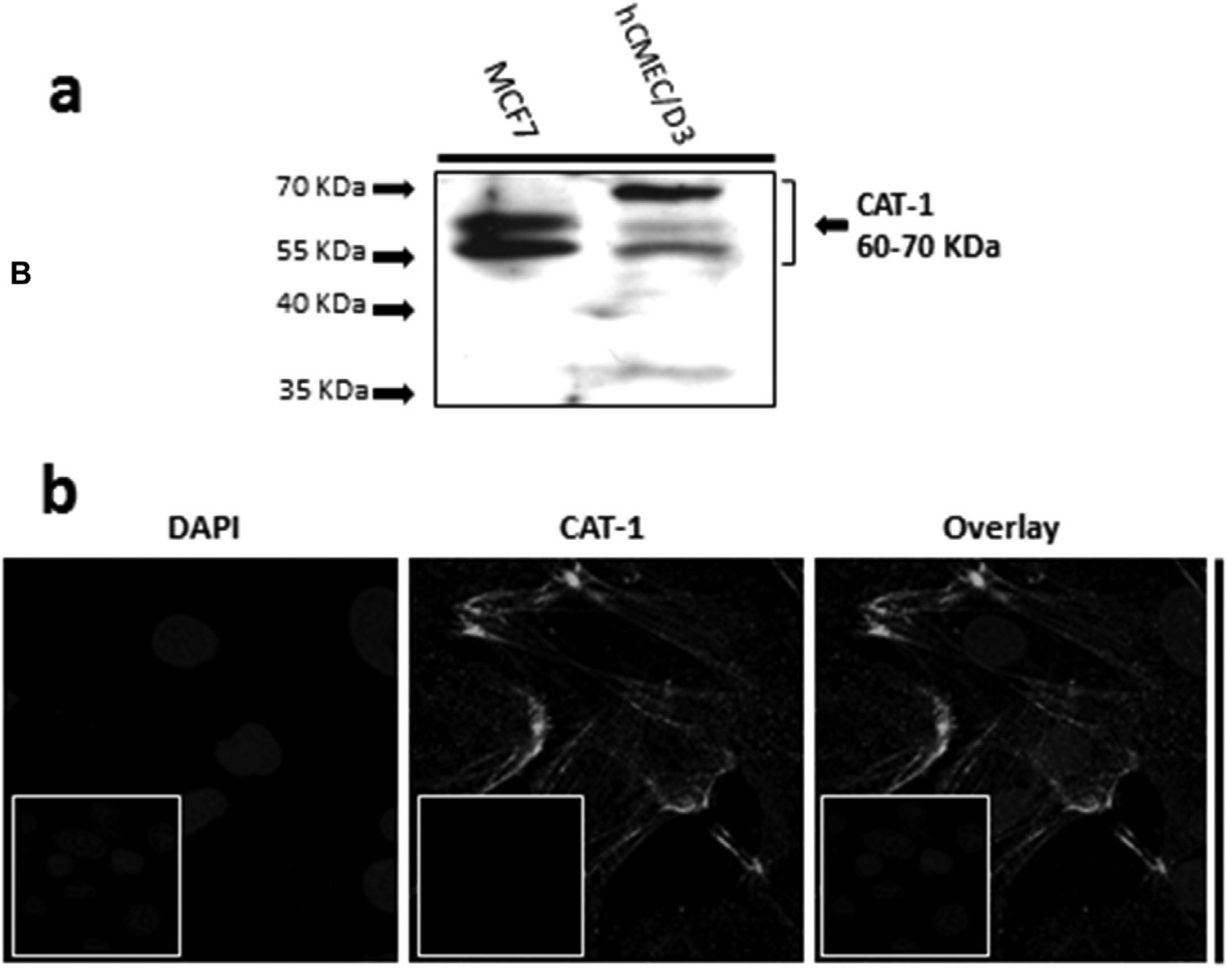
Expression of CAT-1 in hCMEC/D3 cells. (A) SDS-PAGE and WB analysis revealed CAT-1 expression in hCMEC/D3 (P28) and wild type MCF7 whole cell lysate lysed in TGN lysis buffer, as described in Section 4.9. (B) CAT-1 expression was also demonstrated by IF performed with hCMEC/D3 cells (P28) grown on rat tail collagen type 1-coated coverslips, fixed with 4% formaldehyde and stained with primary and secondary antibody, and viewed at 63× with oil emersion using a Zeiss LSM710 confocal microscope and image analysis software Zen 2009 as described in Section 4.9. This localization appears to be on the membrane, similar to the results seen in other studies (Closs et al., 2004). Scale bar, 10 µm. Cell nuclei were counterstained with 1 µg/ml DAPI. For negative staining, cells were stained with secondary antibody only along with DAPI (inserts).

Rabbit anti-human CAT-1 primary antibody was used with goat anti-rabbit HRP conjugated secondary antibody for WB detection of CAT-1 in the TGN lysed hCMEC/D3s and wt MCF7 whole cell lysates. The blot revealed expression of CAT-1 of the expected size of 60–70 kDa (Fig. 3A) according to the antibody manufacturer and previously published studies (Battisti et al., 2012; Mun et al., 2011, Diaz-Perez et al., 2012, Strobel et al., 2012).

CAT-1 expression was also observed with IF and subsequent confocal microscopy in the hCMEC/D3s (Fig. 3B) using rabbit anti-human CAT-1 primary antibody and goat anti-rabbit Alexa Fluor 488 conjugated secondary antibody. It showed typical membrane/cytoskeletal-like patterning (Closs et al., 2004).

### 2.7 Effects of ADMA on hCMEC/D3 permeability to 40 kDa FITC-dextran

**Fig. 4:**
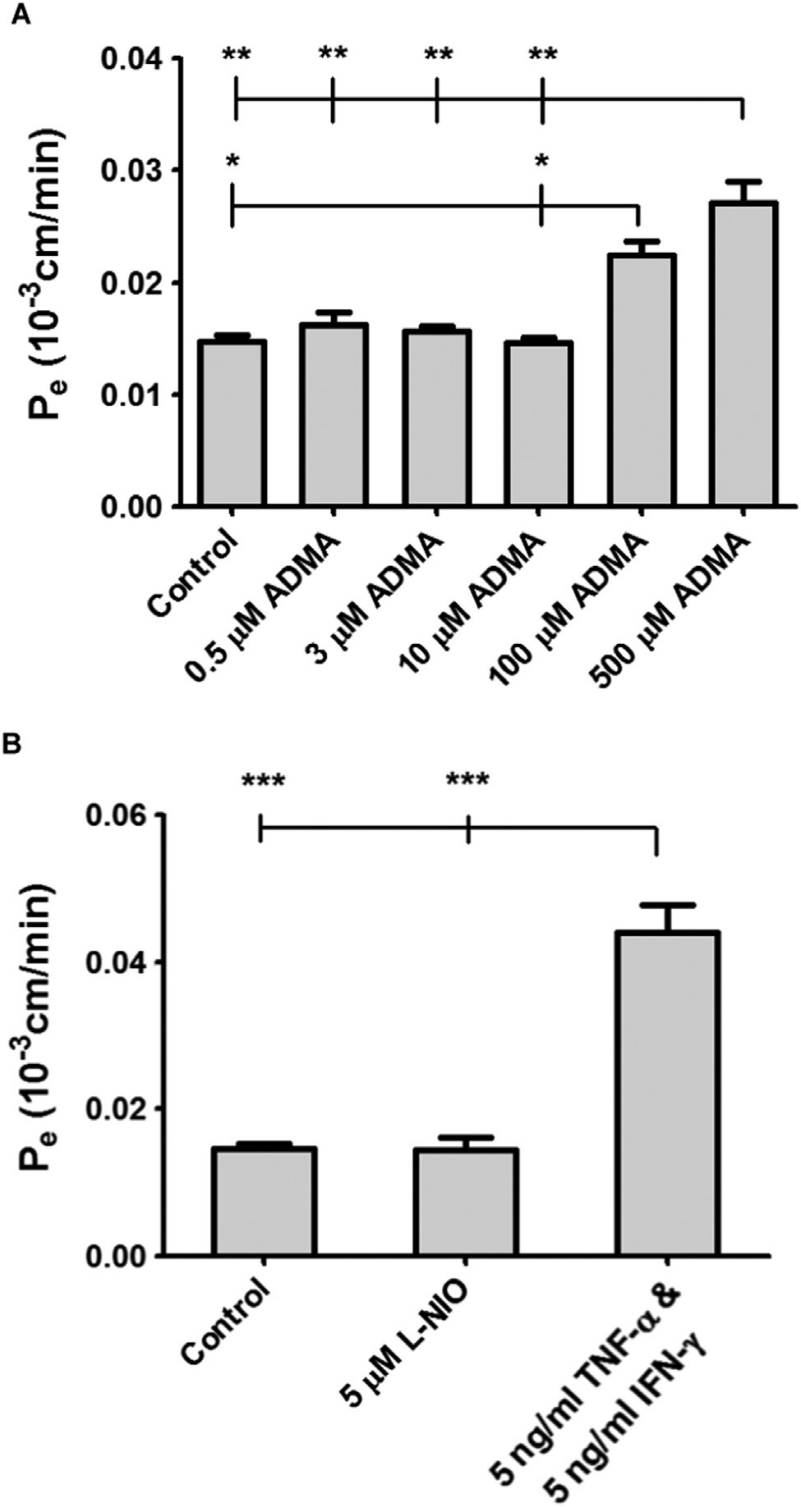
Impact of ADMA, L-NIO, TNF-α and IFN-γ on hCMEC/D3 permeability to 40 kDa FITC-Dex. (A) Confluent monolayers of hCMEC/D3 cells, grown on rat tail collagen type 1-coated transwell filter inserts for 8–10 days, were incubated for 24 hr with a range of ADMA concentrations and their permeability to 40 kDa FITC-Dex was investigated and compared to control (untreated) cells. (B) Confluent monolayers of hCMEC/D3 cells, grown on rat tail collagen type 1-coated transwell filter inserts for 8–10 days, were incubated for 24 h with either 5 µM L-NIO or 5 ng/ml TNF-α & 5 ng/ml IFN-γ, and their permeability to 40 kDa FITC-Dex was investigated and compared to control (untreated) cells. Data represent means±SEM from 12 to 15 transwell filter inserts per treatment. Data analysed with one-way ANOVA and Bonferroni’s post hoc test.

To investigate the impact of ADMA on the integrity of the hCMEC/D3s, cells grown on transwell filters for 8–10 days were incubated with a range of ADMA concentrations for 24 hr before permeability assays with FITC-Dex were performed (Fig. 4A). The concentrations of ADMA used reflected the physiological (0.5 µM), pathophysiological (3 µM) human plasma ranges, as well as 10 µM and 100 µM (values used in published *in vitro* studies by other research groups). 500 µM was used as an extreme reference point. There were no significant differences between the 0.5 µM, 3 µM and 10 µM ADMA treatments between each other or control cells (untreated) on the permeability of the hCMEC/D3 cells to FITC-Dex. However, the incubation of the cells with 100 µM ADMA saw a significant increase in the permeability to FITC-Dex when compared to controls (52% increase, *p*<0.05) and 10 µM ADMA (53% increase, *p*<0.05).

Incubation with 50 µM ADMA caused a significant increase in permeability when compared to the 0.5 µM, 3 µM, 10 µM ADMA treatments and controls (*p*<0.01 in all instances); 84% increase *versus* controls; 67% increase *versus* 0.5 µM ADMA; 73% increase when compared to 3 µM; and an 85% increase compared to 10 µM ADMA. There were no significant differences in permeability coefficients between 100 µM and 500 µM ADMA treatments.

### 2.8 Effects of L-NIO and TNF-α plus IFN-γ on hCMEC/D3 permeability to 40 kDa FITC-dextran

The potent eNOS inhibitor L-NIO and the pro-inflammatory cytokines TNF-α and IFN-γ were incubated with the hCMEC/D3s grown on transwell filter inserts, to investigate their effects on permeability to 40 kDa FITC-Dex.

When compared to controls, 5 µM L-NIO caused no significant changes in permeability (Fig. 4B). When incubated with 5 ng/ml TNF-α and 5 ng/ml IFN-γ, permeability of hCMEC/D3 cells to FITC-Dex significantly increased when compared to controls (*p*<0.001) and L-NIO (*p*<0.001). The percentage increases were 200% compared control and 204% compared to L-NIO.

### 2.9 Cytotoxicity of compounds used

**Fig. 5:**
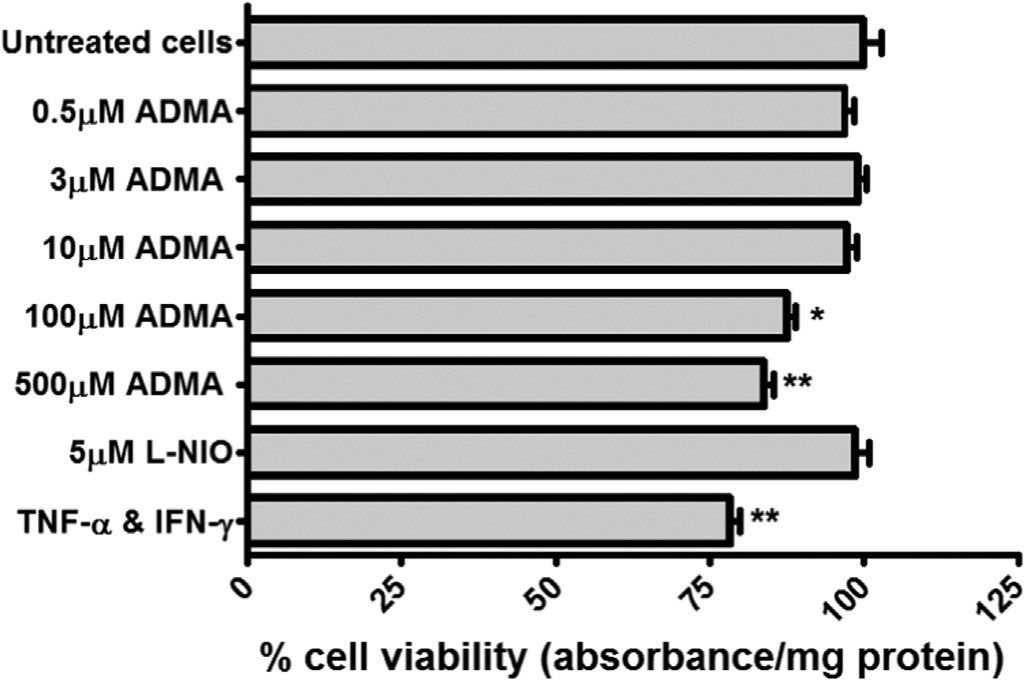
Cytotoxicity of permeability treatments on hCMEC/D3s. The pharmacological treatments used were assessed for any cytotoxic potential using an MTT assay with confluent monolayers of hCMEC/D3 cells in 96 well plates, grown in permeability medium as described in Section 4.3. The results are expressed as percentage viability±SEM and compared to control untreated cells, which were incubated in permeability medium alone. TNF-α and IFN-γ were used together at 5 ng/ml each. N=3 plates, with 6 replicates per plate.

No cytotoxic effects were detected using an MTT assay throughout the experiments, except in the 24-h incubations used in the transwell permeability experiments. Here the ADMA concentrations of 100 µM and 500 µM and 5 ng/ml TNF-α and 5 ng/ml IFN-γ caused significant reductions in cell viability (*p*<0.05*, p*<0.01 and *p*<0.001 respectively for treatments, Fig. 5).

### 2.10 Generation of ROS

**Fig. 6:**
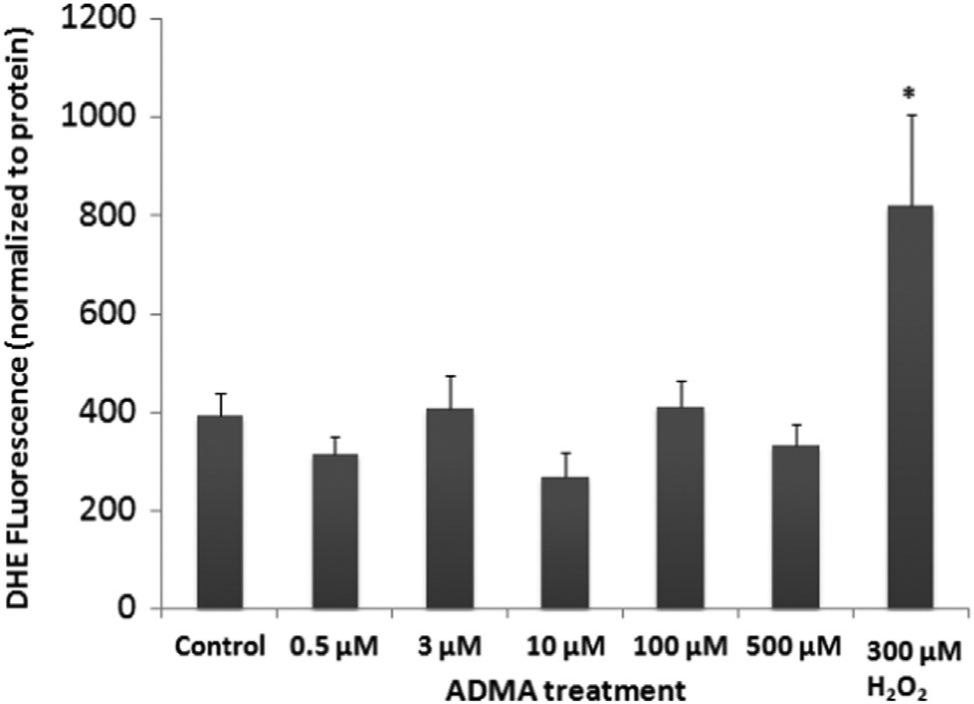
Formation of ROS in hCMEC/D3 cells. hCMEC/D3 cells were treated with increasing doses of ADMA for 24 h before assessment of ROS levels with DHE. As a positive control, some wells were treated with 300 µM H_2_O_2_ alone for 30 min before measuring ROS levels. ADMA treatment had no significant effect on ROS levels in hCMEC/D3 cells, while exposure to H_2_O_2_ significantly increased ROS in these cells (one way ANOVA,*p<0.05).

The effect of ADMA on the generation of ROS was assessed in the cells by measuring DHE fluorescence after 24 h (ADMA) or 30 min (H_2_O_2_) exposure, as described. DHE fluorescence indicates that exposure to ADMA did not increase ROS in these cells after a 24 h exposure at any of the doses tested (Fig. 6). However, 30 min exposure to 300 µM H_2_O_2_ did significantly increase ROS levels in these cells (one way ANOVA, F_6,140_=3.4662, *p*<0.05).

### 2.11 Expression of eNOS and DDAH-1 in hCMEC/D3 cells

To look for expression of the ADMA interacting enzymes eNOS and DDAH-1, SDS-PAGE and WBs were performed on hCMEC/D3 whole cell lysate, with GAPDH as a loading control and HUVEC whole cell lysate used as positive controls. Blots at the expected sizes (140 kDa for eNOS and 37 kDa for DDAH-1) were visible in both the hCMEC/D3s and HUVEC positive controls (data not shown).

## 3 Discussion

The idea that ADMA plays roles in endothelial dysfunction and disease pathogenesis is gaining momentum due to the accumulating *in vitro* and *in vivo* data from a variety of studies on different body regions. ADMA is also a ‘hot-topic’ of investigation in vascular biology research as it is a potent inhibitor of NO production (Teerlink et al., 2009). ADMA is therefore of acute interest to researchers investigating specialised vasculature such as the BBB, particularly given that NO appears to have functional and protective roles as well as deleterious effects depending on its source and the state of brain and cerebrovascular health (Thiel and Audus, 2001). Furthermore, ADMA has been implicated as not only a marker, but also a mediator of cerebral perfusion and cognitive impairment in microangiopathy-related cerebral damage (Kielstein and Kielstein, 2009, Notsu et al., 2009). In acute stroke, increased levels of ADMA have been observed in transient ischemic attacks and cardioembolic infarctions (Wanby et al., 2006, Scherbakov et al., 2012) and ADMA levels in human CSF are believed to be correlated with stroke severity (Brouns et al., 2009). ADMA has also been implicated as a mediator of cerebral vascular tone, by increasing arterial stiffness and decreasing cerebral blood flow independent of blood pressure (Kielstein et al., 2006). We investigated the effects of ADMA on the human BBB by using the hCMEC/D3 *in vitro* BBB model, attempting to shed light on the basic mechanisms expressed at this interface for ADMA.

We first determined the OSPC values for the molecules under investigation, giving us an indication of their lipophilicity and passive membrane penetration without the influence of transporters. The data indicated that both [^3^H]L-arginine and [^3^H]ADMA had high hydrophilicity, with [^3^H]L-arginine having statistically lower lipophilicity than [^3^H]ADMA. Both molecules had higher lipophilic characteristics than [^14^C]sucrose, which is used as an impermeant paracellular permeability and non-specific binding marker in BBB studies. Therefore without the influence of transporters, the intracellular accumulation of [^3^H]L-arginine and [^3^H]ADMA should be low, but in a range similar or higher than [^14^C]sucrose. Interestingly, [^3^H]arginine accumulation into hCMEC/D3 cells was higher than that achieved for [^3^H]ADMA suggesting the influence of transporters.

Our investigations into the interactions between ADMA and CAA transporters is novel with respect to the human BBB and has wider implications due to the ubiquitous expression of CAA transporters throughout the body. Little is known about ADMA transport *in vitro,* especially in human endothelial cell systems mainly due to the assumption that it uses system y^+^ transporters like L-arginine (Teerlink et al., 2009). The only exceptions are studies using human embryonic kidney epithelial cells stably overexpressing CAT1 and CAT2 (2A & 2B) (Strobel et al., 2012, Strobel et al., 2013).

We showed that the accumulation of [^3^H]L-arginine (7nM) was decreased significantly with the addition of 100 µM L-arginine, which is representative of the normal adult L-arginine plasma concentration (Moller et al., 1979, Moller et al., 1983). This suggests transport competition for influx transport mechanisms. Further experiments with 20 mM L-homoarginine revealed that the influx mechanisms utilised was a member of system y^+^, with an almost complete halt of accumulation seen within 1 min (98% decrease) which persisted for the rest of the 30 min time period observed. L-homoarginine is known to competitively inhibit system y^+^ (White, 1985) and the concentration used here was based on that of a previous *in vitro* BBB study using bovine BMECs (O'kane et al., 2006). The impact of L-homoarginine here also dismisses the L-arginine transport roles of other CAA influx mechanisms such as system y^+^L transporters in the transport of L-arginine at the human BBB (O'kane et al., 2006). The lack of inhibition of L-arginine transport upon addition of the NAA leucine at physiological concentrations also supported the finding that system y^+^ appears to be the physiologically relevant transport system used by L-arginine in these cells.

These data were not unexpected, as it has been well established that system y^+^ is the principle CAA transport system in mammals. Which CAT isoform is responsible remains undetermined, but as CAT-1 is well characterised at the BBB, it remains likely that this protein is involved. A recent study investigating ADMA transport showed that CAT2A, CAT2B, and OCT2 indicate a low affinity, high capacity transport for ADMA in a human kidney cell line over-expressing these transport proteins (Strobel et al., 2013), suggesting that these are also involved in BBB transport of ADMA. The expression of CAT-1 was confirmed in hCMEC/D3s cells through WB of whole cell lysates and through IF analysis showing a staining pattern that suggests typical membrane localization and corresponds with previous studies on CAT-1 activity (Closs et al., 2004) as previously demonstrated in pulmonary artery endothelial cells (Mcdonald et al., 1997, Zharikov et al., 2001). We are consequently the first to report expression of CAT-1 in hCMEC/D3 cells, although further research is needed to further confirm the membrane localization of CAT-1 in these cells.

The addition of 500 µM unlabelled ADMA significantly reduced accumulation of [^3^H]L-arginine. In one respect, this finding provides evidence that both molecules can interact with the same transport mechanisms for entry to the cells. On the other hand, although a marked decrease in accumulation was observed, the concentration of 500 µM ADMA is supraphysiological and is not likely to be reached in the body. Importantly, when physiological (0.5 µM) and pathophysiological (3 µM) concentrations of ADMA were used, no significant change in [^3^H]arginine accumulation was observed. A similar finding has been previously reported using human dermal microvascular endothelial cells where 2.5 µM and 10 µM ADMA did not significantly reduce L-arginine accumulation (Xiao et al., 2001).

When investigating the accumulation of [^3^H]ADMA, self-inhibition was time dependent. The addition of 500 µM ADMA produced a significant decrease at all time points (an average of 87%). Overall the self-inhibition data suggests the evidence of influx mechanisms. The fact that the concentration of [^3^H]ADMA used was more than 10 times less than the lowest concentration of added unlabelled ADMA and not greatly affected by the ADMA additions until later time points, suggests that the transporter being used has a low affinity for ADMA and requires a high concentration of unlabelled molecule to demonstrate influx action.

The results from the addition of 100 µM L-arginine show a slight decrease in accumulation at later time points, despite being at a concentration more than 2000 times greater than [^3^H]ADMA. [^3^H]ADMA accumulation was decreased with the addition of 20 mM L-homoarginine or the addition of the NAA leucine at the human physiological plasma concentration (Psychogios et al., 2011). This finding suggests that multiple influx transporters are at work and that influx transport systems separate from system y^+^ could also be transporting ADMA as system y^+^ is insensitive to leucine (Rotmann et al., 2007). The most likely candidates are y^+^L and b^0,+^ proteins, which have affinities for both CAA and NAAs in the presence of Na^+^ ions (O'kane et al., 2006, Rotmann et al., 2007). Both proteins have been demonstrated at mRNA level in hCMEC/D3s cells (Carl et al., 2010).

Based on all these data, we conclude that physiological and pathophysiological extracellular concentrations of ADMA do not compete significantly with L-arginine for entry to cerebral endothelial cells, and that ADMA may use additional transport mechanisms to enter the endothelial cell.

To investigate the effects of ADMA on BBB permeability, we used an assay where hCMEC/D3 cells were grown to confluence on filters and were incubated for 24 h with a range of ADMA concentrations. The P_e_ values obtained were similar to those published with 40–70 kDa FITC-Dextrans for hCMEC/D3 cells (Weksler et al., 2005, Forster et al., 2008). At ADMA concentrations of 100 µM and 500 µM we observed increases in permeability to 40 kDa FITC-Dex when compared to untreated controls. These data are consistent with previous *in vitro* studies investigating ADMA and endothelial dysfunction. In one such study, Chen et al. (2011) used bovine retinal capillary endothelial cells in a permeability model, and incubated the cells with 100 µM ADMA for 24 h and observed an increase in permeability to the paracellular marker, 44 kDa horseradish peroxidise, and a reduced expression of tight junction protein, occludin. In a study by Wojciak-Stothard et al. (2009) porcine pulmonary artery endothelial cells were incubated with 100 µM ADMA for 24 h. They observed a significant increase in permeability compared to control cells (Wojciak-Stothard et al., 2009). Recently, these findings were echoed when ADMA increased the permeability of HUVECs grown on transwell filters to 40 kDa FITC-Dex and FITC-apelin-13 (MW 2053) in a dose-(25–200 µM ADMA) and time- (4, 8, 16 and 24 h) dependent manner (Wang et al., 2011).

Chen et al. (2011) showed that ADMA decreased bovine retinal endothelial cell proliferation in a dose-dependent manner, but did not link these effects as a possible explanation behind the observed increase in permeability. They suggested that ADMA induced ROS formation caused this endothelial dysfunction. Cerebrovasculature (BMECs) are extremely sensitive to oxidative stress due in part to their high expression of NADPH-oxidase, and this makes them vulnerable to increased concentrations of ROS (Uttara et al., 2009, Tripathy et al., 2010). Furthermore, ADMA inhibits the NADPH-oxidase pathway (Wang et al., 2011). In our study, 24 h incubations with 100 µM and 500 µM ADMA decreased hCMEC/D3 cell viability, which may explain the permeability increase. However, the mechanism of this toxicity was not ADMA-induced superoxide formation, as demonstrated by the DHE assay. One way in which ADMA is believed to induce ROS formation is by eNOS uncoupling. With this in mind, we confirmed the expression of eNOS in our cells. The addition of the eNOS inhibitor L-NIO in our permeability experiments however, did not induce any significant permeability differences. Furthermore, DDAH enzymes metabolise ADMA and their activity is significantly decreased in conditions associated with increased oxidative stress such as hyperglycaemia and stroke (Lin et al., 2002, Tain and Baylis, 2007; see Sandoval and Witt, 2008, Chrissobolis et al., 2011 for review). We determined that DDAH-1 was expressed in the hCMEC/D3 cells. Loss of DDAH-1 function in mice has been demonstrated to result in increased ADMA levels and endothelial dysfunction (Leiper et al., 2007). In relation to this, it has been demonstrated that mice aorta and cerebral arterioles over-expressing DDAH-1 are protected against ADMA-induced endothelial dysfunction using 100 µM ADMA (Dayoub et al., 2008).

Interestingly, Wojciak-Stothard et al. (2009) did not find any evidence of ADMA-induced cytotoxicity or changes in ROS in porcine pulmonary artery endothelial cells, but instead showed ADMA-induced cytoskeletal and adherens junction remodelling *via* modulation of the small GTPase Rac1. Small GTPases are implicated in the control of microvascular permeability; with Rac1 a major GTPase required for barrier stabilisation and maintenance (Spindler et al., 2010). Such cytoskeletal remodelling could induce cytotoxic effects explaining the reduced cell viability when the hCMEC/D3s were incubated in high concentrations of ADMA, as discussed in the literature (Stupack and Cheresh, 2003).

TNF-α and IFN-γ increase BMEC permeability through disruption of cell-cell junctions (Minagar and Alexander, 2003) *via* activation of the transcription factor NFκB (Sandoval and Witt, 2008) and tyrosine-phosphorylation of adherens junction proteins by GTPases (Dejana et al., 2008). Their effect on hCMEC/D3 permeability has also been demonstrated (Fletcher et al., 2011) with a similar increase in permeability to FITC-Dex noted (albeit using 70 kDa FITC-Dex). TNF-α and IFN-γ also lead to cytotoxicity in mouse vascular endothelial cells (Yamaoka et al., 2002). It appears likely that in our permeability model a combination of these effects were at work in that the hCMEC/D3 junctional complexes were altered and cytotoxicity-induced apoptosis of the cells contributed to the increase in permeability.

The results of this study are twofold: firstly, they demonstrate that both L-arginine and ADMA appear to use system y^+^ influx mechanisms to enter hCMEC/D3 BBB cells, as predicted due to their close structural similarity and chemical composition. They also demonstrate that this transporter could be CAT-1, the expression of which was confirmed in our human BBB model. Other influx systems such as systems y^+^L and b^0,+^ appear to have smaller roles in L-arginine transport, but may have more important roles for ADMA influx. Also physiological and pathophysiological extracellular concentrations of ADMA do not compete significantly with L-arginine transport into the BBB *via* system y^+^. Secondly, the data from our study show that ADMA is able to increase paracellular permeability in human BBB cells. Although it was high ADMA concentrations that induced these permeability increases, that does not rule out the role of more clinically relevant concentrations *in vivo,* especially considering the sensitivity of intracellular DDAH enzymes to oxidative stress which can be induced from several sources during various disease states. ADMA has been demonstrated to induce endothelial dysfunction that could have serious implications in brain and cerebrovasculature pathologies, as well as on the integrity of the BBB itself. Consequently, ADMA is an interesting therapeutic target in neuro-inflammatory diseases. The fact that our BBB model expressed both eNOS and DDAH-1 makes it a good human *in vitro* BBB model to study the effects of ADMA and NO inhibition now and in future studies.

## 4 Experimental procedure

### 4.1 Materials

Tritium labelled L-arginine was purchased from American Radiolabelled Chemicals, Inc., MO, US and had a specific activity of 43 Ci/mmol. [^14^C]sucrose was purchased from Moravek Biochemicals and had a specific activity of 4980 mCi/mmol. [^3^H]ADMA was custom radiolabelled by GE Healthcare, Amersham, UK and had a specific activity of 8 Ci/mmol. The Pierce bicinchoninic acid (BCA) protein assay, enhanced chemiluminescence (ECL) kit and all culture wear was purchased from Thermo Scientific, Thermo Scientific Nunclon, Loughborough, UK. The EGM-2MV BulletKit was purchased from Lonza, Wokingham, UK. Rat tail collagen type-I, penicillin-streptomycin, 0.25% Trypsin-EDTA, recombinant human TNF-α, recombinant human IFN-γ, Dulbecco’s modified Eagle's medium without phenol red (DMEM) and Dulbecco's Phosphate-Buffered Saline without calcium, magnesium and phenol red (DPBS) were all purchased from Gibco, Invitrogen, Paisley, UK. Foetal bovine serum (FBS) ‘gold’ was purchased from PAA the Cell Culture Company, Yeovil, Somerset, UK. Dimethyl sulfoxide (DMSO), phosphate buffered saline (PBS), 1 M HEPES, (3-(4,5-Dimethylthiazol-2-yl)-2,5-diphenyltetrazolium bromide (MTT), unlabelled ADMA, unlabelled L-arginine, L-homoarginine, L-leucine, 40 kDa fluorescein isothiocyanate labelled dextran (FITC-Dex), Triton x-100, Hank's balanced salt solution (HBSS), TGN lysis buffer components (Tris, NaCl, 10% glycerol, glycerophosphate B, Tween-20 and NP-40), β-mercaptoethanol, ammonium persulfate (APS), *N*,*N*,*N*′,*N*′-tetramethylenediamine (TEMED), 1-octanol, dihydroethidium (DHE), hydrocortisone, ascorbic acid, bFGF and Corning® Transwell® polyester membrane inserts (pore size 0.4 µm, membrane diameter 1.12 cm^2^) were all purchased from Sigma Aldrich Company Ltd., Poole, UK. *N*^5^-(1-Iminoethyl)-L-ornithine (L-NIO) was manufactured by Cayman Chemicals and purchased from Cambridge Bioscience Ltd., Cambridge, UK. Proteoblock™ protease inhibitor cocktail and Page Ruler Prestained 10–170 kDa Protein Ladder were purchased from Fermentas Life Sciences, North Yorkshire, UK. 0.45 µm Immobilon-P polyvinylidene fluoride (PVDF) membrane was purchased from Millipore UK Ltd., Watford, UK. Human umbilical vein endothelial cell lysate was purchased from Santa Cruz Biotechnology, Inc., California, US. Wild type MCF-7 whole cell lysate was kindly provided by Dr. Evangelos Pazarentzos of Imperial College London. Rabbit anti-human SLC7A1/CAT-1 polyclonal primary antibody, goat anti-human DDAH1 polyclonal primary antibody, mouse anti-human GAPDH monoclonal primary antibody, rabbit anti-goat HRP conjugated polyclonal secondary antibody, goat anti-rabbit HRP conjugated polyclonal secondary antibody and rabbit anti-mouse HRP conjugated polyclonal secondary antibody were all purchased from Abcam, Cambridge, UK. Rabbit anti-human NOS3/eNOS polyclonal primary antibody was purchased from BD Biosciences, VWR, Leicestershire, UK. 4′,6-diamidino-2-phenylindole (DAPI) was purchased from New England Biolabs, Bristol, UK. Goat anti-rabbit Alexa Fluor 488 conjugated secondary antibody was purchased from Invitrogen, Paisley, UK. The hCMEC/D3 cell line was obtained from Professor Pierre O. Couraud (Institut Cochin, Université Paris Descartes, CNRS, Paris, France), Professor Babette Weksler (Weill Medical College of Cornell University, New York, NY, USA) and Dr Ignacio Romero (The Open University, Department of Life Sciences Walton Hall, Milton Keynes, UK). Human umbilical vein endothelial cell (HUVEC) whole cell lysate was purchased from Santa Cruz Biotechnology, Inc., California, US. MCF7 whole cell lysate was a kind gift from Professor Stefan Grimm (Imperial College London).

### 4.2 Cell culture

The hCMEC/D3s were cultured using EGM-2MV BulletKit as described previously (Poller et al., 2008, Watson et al., 2012). All cells used in the experiments were between passage 25 and 35 and cells used in accumulation experiments were seeded at 2.5×10^4^ cells/cm^2^ on 0.1 mg/ml rat tail collagen type-I in HBSS.

Cells grown on transwell filters (Corning Transwell polyester membrane inserts, pore size 0.4 µm, membrane area 1.12 cm^2^) were seeded at a density of 120,000 cells per filter (determined using a haemocytometer). Filters were pre-coated for 2 h with 0.15 mg/ml rat tail collagen type-I in HBSS, before being washed once with DPBS at 37 °C and then incubated in permeability media before seeding (0.5 ml in donor well/filter insert and 1.5 ml in receiver wells).

The permeability media is a reduced growth factor media which has been optimised by Professor Pierre Couraud's group (Institut Cochin, Université Paris Descartes, CNRS, Paris, France) and follows an established protocol allowing the cells to differentiate and form restrictive tight junctions, restricting paracellular permeability (Poller et al., 2008; Zougbede et al., 2011). Permeability media consisted of EBM-2 endothelial basal medium from the Clonetics® EGM®−2MV BulletKit® with 5% FBS ‘gold’, 1% penicillin-streptomycin, 1.4 μM hydrocortisone, 5 μg/ml ascorbic acid, 10 mM HEPES and 1 ng/ml bFGF. The cells were also grown in an incubator with a saturated humidity at 37 °C in 5% CO2 and 95% fresh air. Media was changed every 2–3 days and cells used for experiments were between 8 and 10 days post-seeding.

Protein expression (BCA protein assay) and integrity of plasma membranes ([^14^C]sucrose) were monitored to confirm cell viability and used for correction factors (see details below).

### 4.3 Accumulation assays

For accumulation experiments, cells were grown to 100% confluency (reached at 4 days) in collagen coated 96 well plates and then left for a further 3 days until experiments (7 days after seeding). Medium was changed every 2–3 days. Experiments were performed on confluent monolayers of hCMEC/D3s, grown in the centre 60 wells of 96 well plates. Medium was aspirated from wells and replaced with a 200 μl aliquot of [^3^H]L-arginine or [^3^H]ADMA (7 nM or 38 nM, respectively) with [^14^C]sucrose (972 nM) as a correction for non-specific binding, with or without transporter interacting substrates/inhibitors in accumulation assay buffer (the composition of which has been published previously) (Watson et al., 2012).

Cells were exposed to the [^3^H]test molecule/[^14^C]sucrose/buffer mix for five different time periods (1, 2.5, 5, 20 and 30 min) allowing assessment of radiolabelled molecule accumulation in the cells. The accumulation assays were performed on a temperature-controlled shaker (THERMOstar, BMG labtech, Offenburg, Germany) at 37 °C and 120 rpm. Once each column of cells had been exposed for the correct amount of time, the wells were washed 3 times with ice-cold phosphate buffered saline (1× PBS, Gibco, Invitrogen, UK) to stop transport processes and remove radiolabelled test molecules and buffer that had not accumulated in the cells. The cells were then lysed by adding 200 μl of 1% Triton x-100 (Sigma, UK) per well for 1 h at 37 °C to liberate any accumulated radiolabelled test molecule and solubilise the cell proteins. 100 μl of each well was then added to scintillation vial along with 4 ml scintillation fluid (Optiphase Hisafe 2, PerkinElmer, UK) added and samples counted on a Tricarb 2900TR liquid scintillation counter (μl/mg). The remaining 100 μl in each well was used in a BCA™ protein assay, using bovine serum albumin as standards, and protein concentration measured spectrophotometrically on a Labsystems Multiscan reader with Ascent software. Total accumulation of [^3^H]test molecule in the cells was expressed as a volume of distribution (V_d_). V_d_ is derived from the ratio of dpm/mg protein in the lysate to dpm/μl of the accumulation buffer. The V_d_ values for [^3^H]test molecule were corrected with the V_d_ values for [^14^C]sucrose which is a marker of non-specific binding and extracellular space.

### 4.4 Transporter inhibition assays

For [^3^H]L-arginine self-inhibition experiments, (which provide evidence of saturable mechanisms), 100 μM unlabelled L-arginine (a similar concentration to that present in human plasma) was added to the accumulation assay buffer along with the [^3^H]L-arginine and [^14^C]sucrose. In a separate experiments, the system y^+^ competitive substrate L-homoarginine (20 mM) and L-leucine (100 μM) were individually added to the accumulation assay buffer to investigate the role of system y^+^ and system y^+^L transporters on the transport of L-arginine.

In a separate series of accumulation experiments, the effects of a range of ADMA concentrations on [^3^H]L-arginine accumulation were also assessed. The physiological plasma concentration of 0.5 μM, the pathophysiological plasma concentration of 3 μM and the supraphysiological concentration of 500 μM (as an extreme reference point) were added separately to the accumulation buffer alongside [^3^H]L-arginine and [^14^C]sucrose (Teerlink et al., 2009).

For [^3^H]ADMA investigations, evidence for influx transport activity was assessed by self-inhibition studies using 0.5 μM, 3 μM and 500 μM unlabelled ADMA alongside [^3^H]ADMA and [^14^C]sucrose in the accumulation buffer. The influence of 100 μM unlabelled L-arginine, 100 μM L-leucine and 20 mM L-homoarginine were also separately investigated as with [^3^H]L-arginine.

### 4.5 L-arginine and ADMA Octanol-saline partition coefficient (OSPC)

To gain an indication of the lipophilicity of the study molecules [^3^H]ADMA and [^3^H]L-arginine, an OSPC was performed using 0.037 MBq (1 μCi) of each molecules in 1 ml of saline as described previously (Sanderson et al., 2008). The OSPC of [^14^C]sucrose has been previously published by our group (Jeganathan et al., 2011). All OSPC measurements were performed in triplicate.

### 4.6 Permeability assay

To investigate the effect of ADMA on the integrity of the hCMEC/D3s, the paracellular permeability was investigated using permeability assays on confluent monolayers of hCMEC/D3s grown on 12 well plate transwell filter inserts.

At 8–10 days post seeding, the TEER of the monolayers on the filters was measured as described previously (Patabendige et al., 2012), at least 4 h before the assay. Filters with TEERs less than the published hCMEC/D3 TEER values were not used for the permeability assays (Poller et al., 2008, Fletcher et al., 2011). Permeability media was replaced 24 h before the assay in both the insert (0.5 ml) and donor wells (1.5 ml) with fresh media containing one of the following concentrations: 0.5 µM ADMA, 3 µM ADMA, 1 µM ADMA, 100 µM ADMA or 500 µM ADMA. In a separate series of experiments, media was added to the cells for 24 h containing either L-NIO (a non-selective inhibitor of NOS isoforms,) at a concentration of 5 µM (which is within the 3.9 µM K_i_ range of eNOS) (Rees et al., 1990), or 5 ng/ml recombinant human TNF-α with 5 ng/ml recombinant human IFN-γ, which have been show to increase brain microvascular endothelial cell (BMEC) permeability (Minagar and Alexander, 2003, Sandoval and Witt, 2008, Dejana et al., 2008).

On the day of the experiment, this media was removed from the transwell filter insert and the donor well, and 1.5 ml pre-warmed (to 37 °C) DMEM (without phenol red)+2% FBS was added to the wells of fresh 12 well plates. 40 kDa FITC-Dex was diluted to 2 mg/ml in a separate vial of DMEM +2% FBS assay media and used as a BBB paracellular marker for the assay (Hoffmann et al., 2011) by adding 0.5 ml of it to the transwell filter insert only.

The filter was then moved into a new donor well on flat bottom 12 well plates (containing DMEM +2% FBS) at 10-min intervals for 60 min. The fluorescence in each well of 12 well plate was then measured in a FlexStation® 3 Bench top Multi-Mode Microplate fluorescent plate reader at an excitation wavelength of 485 nm and emission wavelength of 530 nm, using SoftMax® Pro Microplate Data Acquisition & Analysis software.

Permeability of FITC-Dex during the assay was determined as permeability coefficients (Pe) which take into account the relation between the permeability of the monolayer and the permeability of empty filter pre-coated with rat tail collagen type 1 (without cells). These calculations are based on work by Dehouck et al. (1992) and Cecchelli et al. (1999), and have been used in subsequent hCMEC/D3 publications (Weksler et al., 2005, Poller et al., 2008, Tai et al., 2009).

### 4.7 Detection of ROS formation

hMEC/D3 cells were plated into collagen-coated 24 well plates and grown for 3 days. Cells were treated with either media alone (control) or media containing ADMA (0.5, 3, 10, 100 and 500 µM) or 300 µM hydrogen peroxide (H_2_O_2_) as a positive control for 24 h. At the end of the incubation time, media was removed and cells were incubated in 10 µM of the fluorescent probe DHE, which detects intracellular •O_2_^.−^ production (D'agostino et al., 2007) in PBS for 30 min at 37 °C. Cells were washed in PBS and fixed using freshly prepared 4% paraformaldehyde. After fixation, cells were washed and stored in PBS at 4 °C for 3–5 days until fluorescent analysis was performed on a BioTek Synergy HT multi-mode microplate reader using 530 nm/590 nm excitation and emission filters.

### 4.8 MTT assay

The cytotoxic effects of all the test molecules used in this study were assessed on confluent monolayers of cells in 96 well plates using an MTT assay described previously (Watson et al., 2012). Absorbance values were corrected by protein content (determined using a BCA assay) and expressed as percentage viability compared to control untreated cells.

### 4.9 DDAH-1, eNOS and CAT-1 expression studies

To investigate the expression of eNOS and DDAH-1 in the hCMEC/D3s, SDS-PAGE and Western blotting (WB) were performed on whole cell lysates, prepared in TGN lysis buffer as described previously (Watson et al., 2012). Briefly, lysates were prepared from cells grown for 8 days in permeability medium and 30 μg of each lysate was loaded per well on a 10% SDS-PAGE gel. Following electrophoresis, proteins were transferred using semi-dry transfer onto methanol activated Immobilon-P PVDF membranes (0.45 µM pore size), blocked for 2 h at RT in PBS-Tween (PBS-T) with 5% milk powder and incubated overnight at 4 °C with goat anti-human DDAH-1 primary antibody at a dilution of 0.2 μg/ml PBS-T with 0.5% BSA, or Rabbit anti-human NOS III/eNOS primary antibody, used at a dilution of 1:2000 in PBS-T and 0.5% BSA. Membranes were then washed 3× with PBS-T and incubated for 1 h at RT with rabbit anti-goat HRP conjugated secondary antibody was used at a dilution of 1:2500 in PBS-T with 0.5% BSA for chemiluminescence detection of DDAH-1 and goat anti-rabbit HRP conjugated secondary antibody was used at a dilution of 1:5000 in PBS-T with 0.5% BSA for chemiluminescence detection of eNOS. GAPDH was probed as a loading control using mouse anti-human primary antibody at 1:2000 in PBS-T with 0.5% BSA, and rabbit anti-mouse HRP conjugated secondary at 1:2000 in PBS-T with 0.5% BSA was used for chemiluminescence. For eNOS and DDAH-1 expression, and HUVEC whole cell lysate in RIPA buffer was used as a positive control (Murakami et al., 2006; Zhang et al., 2011).

The expression of CAT-1 was determined by SDS-PAGE, WB, and immunofluorescence (IF) with confocal microscopy. For SDS-PAGE and WB, the procedure above was used and immobilised proteins probed with rabbit anti-human CAT-1 primary antibody at a dilution of 1:250 in PBS-T and 0.5% BSA. Goat anti-rabbit HRP conjugated secondary antibody was used at a dilution of 1:5000 for chemiluminescence detection of CAT-1. Whole wild-type MCF7 cell lysate, prepared in TGN lysis buffer, acted as positive controls for this transporter (Abdelmagid et al., 2011).

IF of CAT-1 was performed on hCMEC/D3s grown on rat tail collagen type 1 coated sterile glass coverslips using a previously described protocol (Watson et al., 2012). Goat anti-rabbit Alexa Fluor 488 conjugated secondary antibody diluted at 1:200 in PBS was used to stain CAT-1 bound primary antibody used at a dilution of 1:200 in PBS-T overnight on the fixed hCMEC/D3s. 0.1 µg/ml DAPI was used to stain cell nuclei before mounting. Slides were viewed with a Zeiss LSM710 confocal microscope and image analysis software Zen 2009 at 63x (Zeiss, Germany).

### 4.10 Statistical analysis

Comparisons were made between control and test plates of cells and differences at *p*<0.05 considered significant. Multiple-time accumulation data were analysed by Two Way Repeated Measures ANOVA tests and Holm-Sidak posthoc tests. MTT assay and permeability assay data and ROS formation were compared to controls using One Way ANOVA and Bonferonni post-hoc tests. All data were analysed using Sigma Plot version 11.0 software (SPSS Science Software UK Ltd., Birmingham UK) and expressed as mean±SEM.

## Conflicts of interest

The authors acknowledge that there are no conflicts of interest.
